# Supplementary material for: A cloud-based workflow to quantify transcript-expression levels in public cancer compendia
Source: Sci Rep. 2016 Dec 16;6:39259. doi: 10.1038/srep39259 (PMC5159871; doi:10.1038/srep39259)
Supplement: Supplementary Materials [file srep39259-s1.pdf]

Supplementary Material

A cloud-based workflow to quantify transcript-expression levels in public cancer compendia

PJ Tatlow[1], Stephen R. Piccolo[1,2,*]

1 - Department of Biology, Brigham Young University, Provo, Utah, USA

2 - Department of Biomedical Informatics, University of Utah, Salt Lake City, Utah, USA

* - Correspondence should be addressed to S. R. P. (801-422-7116; stephen_piccolo@byu.edu).

Supplementary Figures

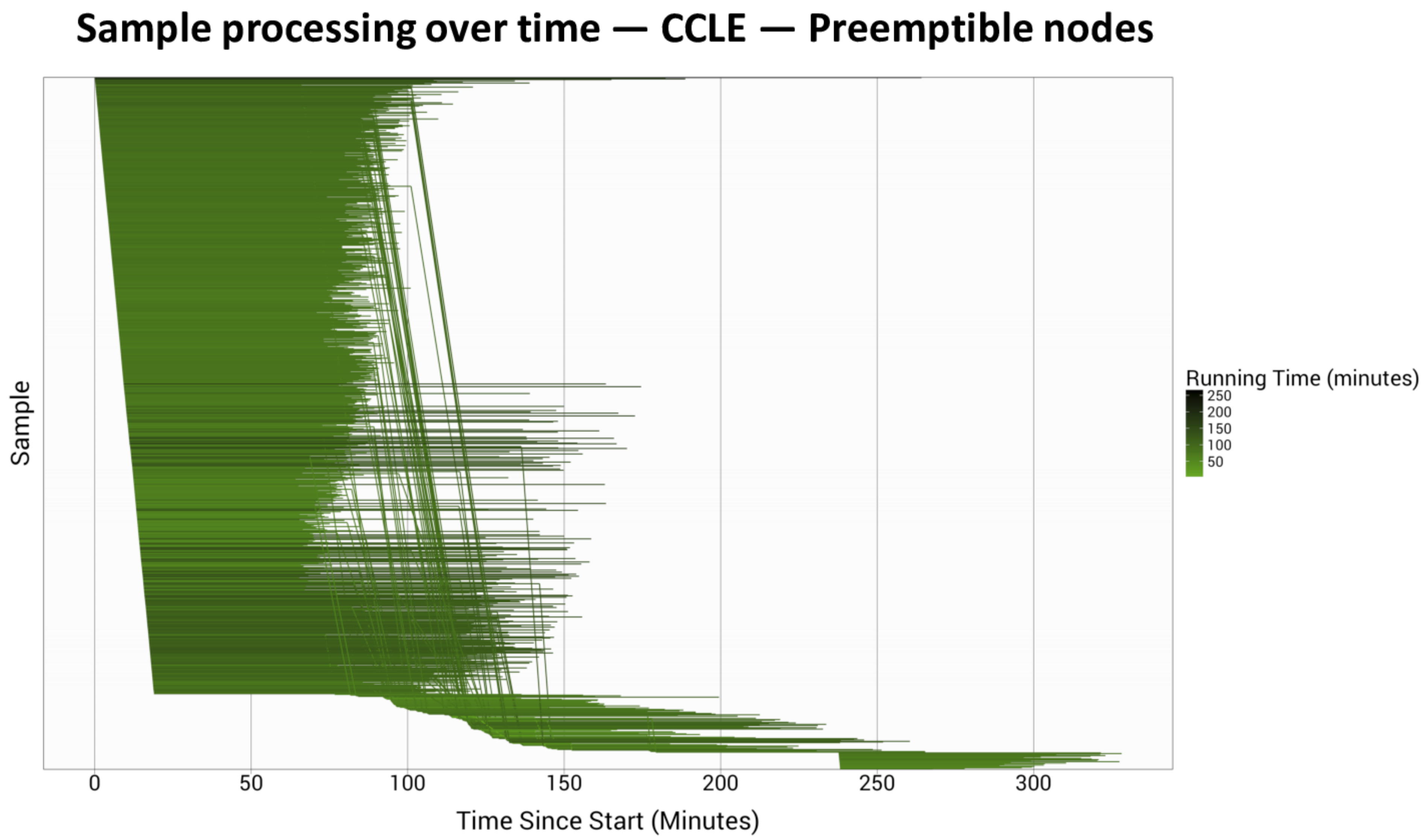

Figure S1. Processing time per CCLE sample using the preemptible-node configuration. The 934 CCLE samples were processed using a variable number of preemptible virtual machines. The horizontal lines represent the relative start and stop times at which each sample was processed. Darker lines identify samples that took longer to process. Vertical lines indicate times at which samples were preempted and then resubmitted for processing. In total, 78 preemptions occurred.

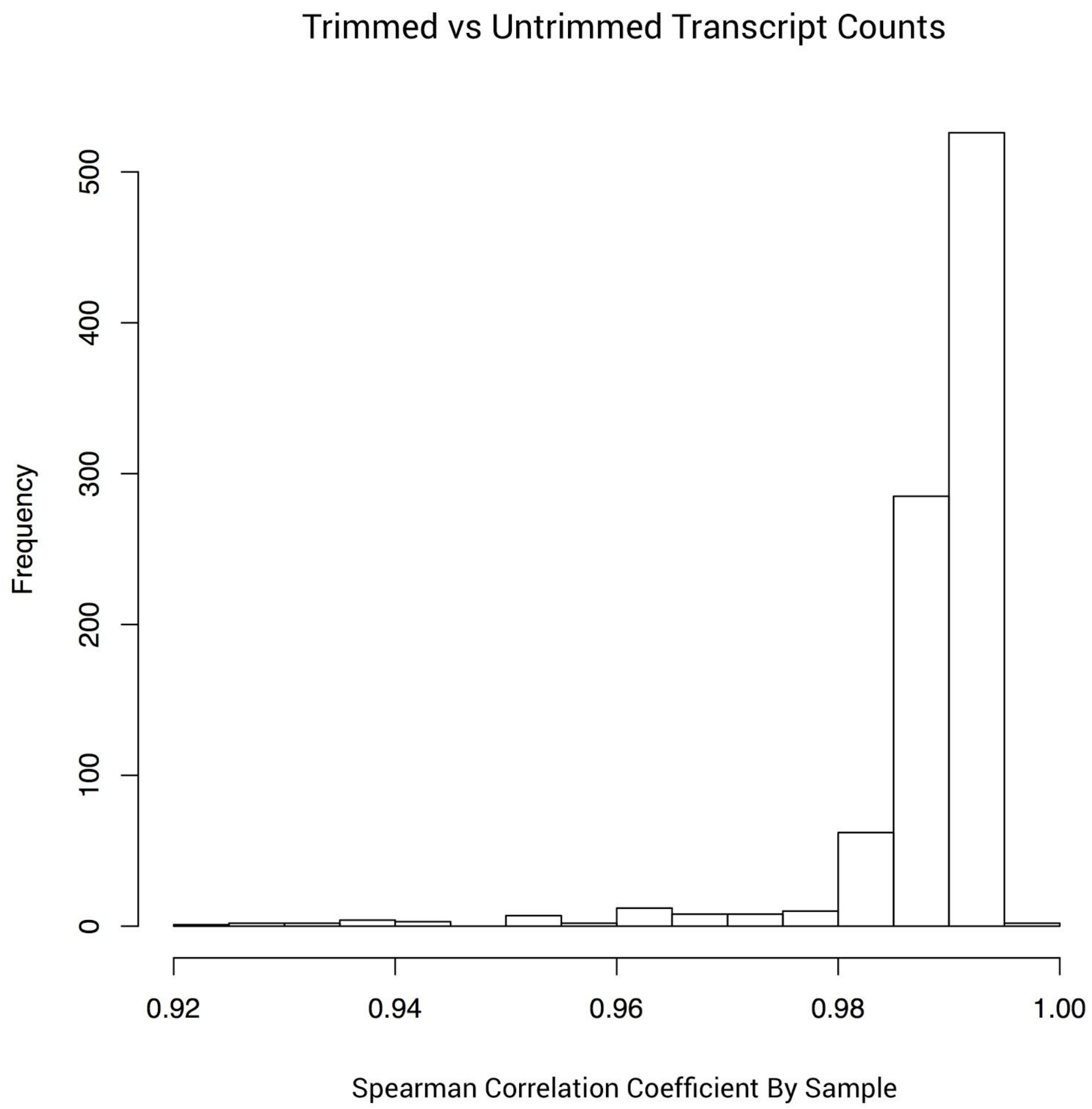

Figure S2. Histogram of Spearman correlation coefficients comparing read counts for trimmed vs. untrimmed CCLE data.

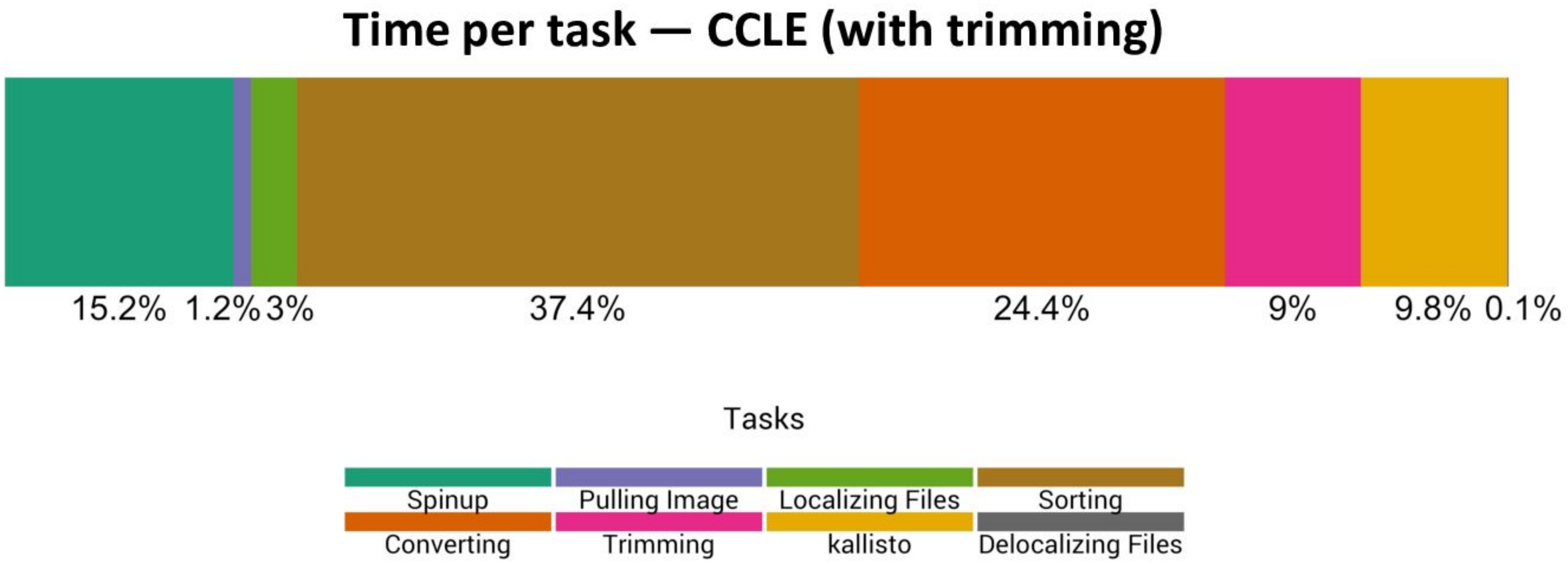

Figure S3. Relative time spent on computational tasks for CCLE samples using the preemptible-node configuration. We logged the durations of individual processing tasks for the CCLE samples, averaged these values, and calculated the percentage of overall processing time for each task. The “spinup,” image pulling, and file localization steps enabled the virtual machines to begin executing. The “spinup,” image pulling, and file localization steps enabled the virtual machines to begin executing. For sample preprocessing, the BAM files were sorted, converted to FASTQ format, and trimmed for quality; these steps took 61.8% of the overall processing time. The *kallisto* alignment and quantification steps took only 9.8% of the overall processing time.

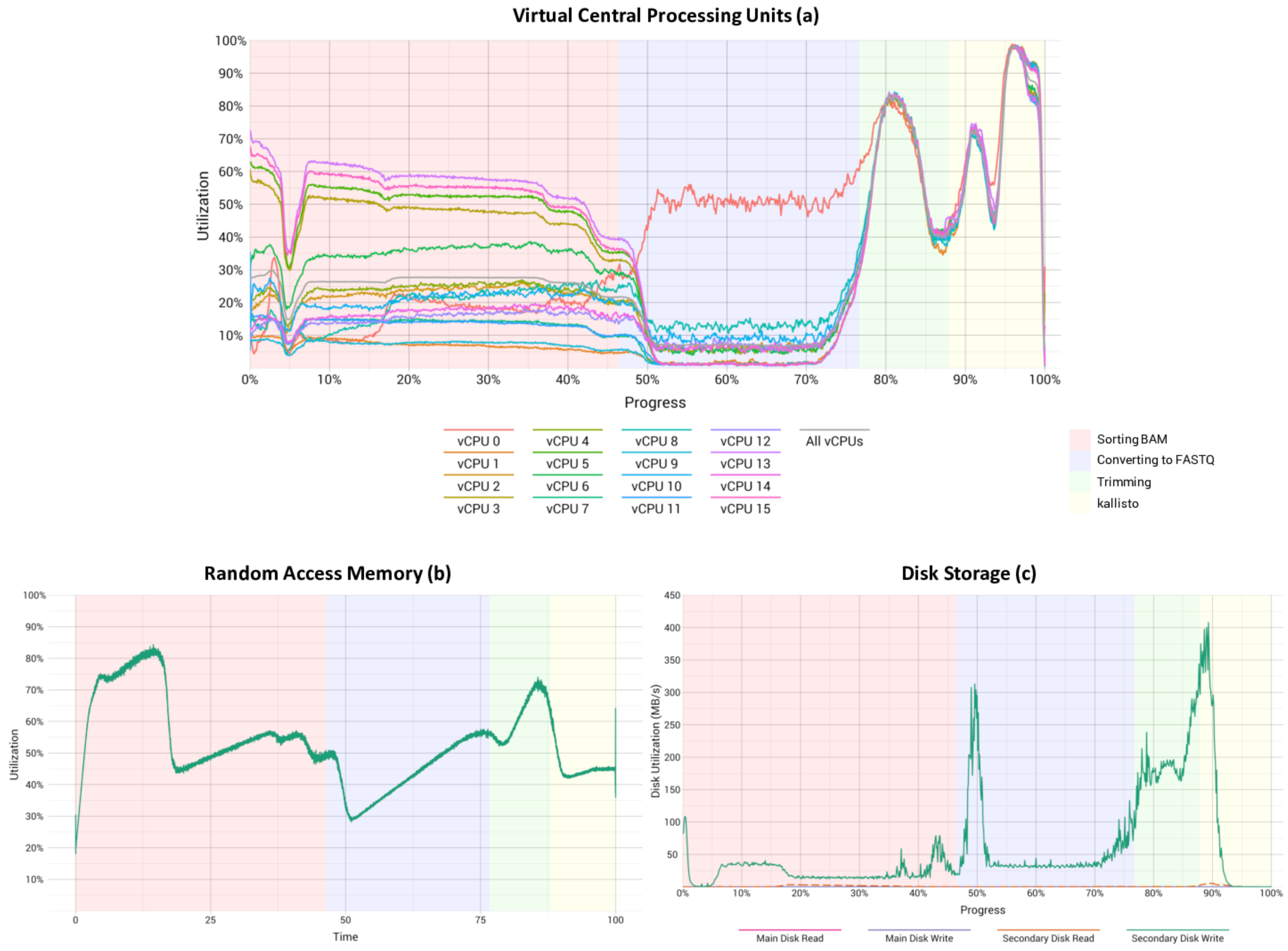

Virtual Central Processing Units (a)
Utilization
Progress
vCPU 0
vCPU 1
vCPU 2
vCPU 3
vCPU 4
vCPU 5
vCPU 6
vCPU 7
vCPU 8
vCPU 9
vCPU 10
vCPU 11
vCPU 12
vCPU 13
vCPU 14
vCPU 15
All vCPUs
Sorting BAM
Converting to FASTQ
Trimming
kallisto
Random Access Memory (b)
Utilization
Time
Disk Storage (c)
Disk Utilization (MB/s)
Progress
Main Disk Read
Main Disk Write
Secondary Disk Read
Secondary Disk Write

Figure S4. Computational resource utilization while CCLE samples were processed using a preemptible-node configuration. These graphs show the (a) percentage of user and system vCPU utilization, (b) percentage of memory usage, and (c) disk activity. The “main” disks had only 10 gigabytes of storage space and stored operating-system files. The “secondary” disks, which stored all data files, had 350 gigabytes of space. The background colors represent the computational tasks shown in Figure S3 and correspond with expected resource utilization for these tasks. (We were unable to collect performance metrics for preliminary tasks, such as file localization, because these tasks were not performed within the software container.) Each graph summarizes data from all 934 CCLE samples.
